# Abstract of the Proceedings of the Buffalo Medical Association

**Published:** 1855-12

**Authors:** Sanford B. Hunt


ART. IV.—*Abstract of the Proceedings of the Buffalo Medical Association.*

Tuesday Evening, Nov. 6, 1855.

Association met.

Present—Drs. Newman, Wilcox, Mixer, Burwell, Almy, Baker, Wyckoff, Rochester, Hamilton, Samo, Hunt, Lay, Strong, White, Eastman, Hawley, Treat, Gould, and Howell.

The President, Dr. Strong, being absent at the opening, Dr. Hamilton was appointed chairman *pro tem*, but Dr. Strong coming in subsequently, Dr. Hamilton resigned the chair.

The minutes of the preceding meeting were read. The following corrections were made: In the prescription mentioned by Dr. White, reverse the quantities of sulph. ferri and iodid. potassii, so as to read *sulph. ferri* ʒiv., iodid. potassii ʒij. Dr. Wyckoff also corrected his case of fracture of the clavicle. Instead of being called on the first day after the accident several days had elapsed.

Dr. J. N. Brown was elected a member of the association.

*Dr. Baker* reported from the committee on the subject of procuring a charter that the association can incorporate easily and at small cost. It remained for the association to decide on the propriety of so doing.

*Dr. Wilcox* moved to accept the report.

*Dr. Newman* moved to amend by laying on the table. He wished to leave the labor of organization in the hands of the present committee, and thus save the appointment of a new one.

*Dr. Wilcox* accepted the amendment, and the motion as amended passed *nem. con.*

*Dr. Burwell* then read the following paper on pneumonia:

I respectfully ask the attention of the society to the histories of two cases of sthenic pneumonia, as follows:

CASE I. Charles Harper, aged five years and ten months, of slight frame for one of his age, and of general good health, was taken sick on the afternoon of Oct. 1st, last. No cause could be given for his sickness. He got feverish without any chill. He had a high fever all that night and complained of pain in his bowels. Took physic Tuesday morning (Oct. 2d) which operated three or four times. I saw him first that night. He had then a dry, burning skin, a highly flushed countenance, rapid breathing and pulse, great thirst and a short, hacking cough.

Prescribed antimony, to be given until vomiting or purging supervened.

Oct. 3, 8, P. M. No amelioration of symptoms whatever, although he has been vomited freely and repeatedly by the antimony. Coughs more, and complains of pain low down in the right side in the region of the liver. Pulse 150, full and hard; respiration 60. I took four ounces of blood from the arm. It produced palor of the face, but did not sensibly affect the hardness of his pulse. The blood had a firm clot, but was without a buffy coat.

To continue the antimony, but not in doses to vomit him. To take also some small powders of Dov. powder and calomel.

Thursday, Oct. 4, 8, A. M. The high febrile symptoms of yesterday lasted all night, without the least apparent abatement, as a consequence of the bleeding. But about 7 o'clock this morning, he became much paler and broke out into a light sweat. Pulse 120; respiration 60. There has not been, until now, any physical sign of pneumonia. This morning there is a dryness, or clearness, or a higher pitch, however it may be described, of the respiratory murmur at the root of the right lung.

8, P. M. The fever has been quite moderate to-day, until about 4, P. M., when it got higher. Pulse now 120; respiration 56; cheeks flushed; marked palor around nose and mouth. Complains to-night of pain in the right shoulder.

There is to-night, for the first time, a distinct bronchial respiration at the root of the right lung. It extends downward toward the base of the chest, but not into the axillæ. No crepitus. A coarse crackle of a thick mucous rhonchus heard occasionally.

Prescribed Dov. powder in a large dose, to procure quietness, and antimony regularly every hour.

Oct. 5, Friday, 8, A. M. Rested well last night with one Dov. powder only. He is pale, has a moderately warm skin; had some fever all night. Pulse 120, active; respiration 52, catching. Had quite a severe pain in right side for an hour this morning. Coughs more; it is quite loose. The bronchial respiration distinct in middle lobe, right lung, posteriorly, and faint in the lower lobe. No crepitus; has the mucous rattle. Percussion quite dull over the inflamed lobes. Bowels have moved twice this morning.

Continue the antimony.

4, P. M. Find both cheeks highly flushed, and the white circle around the nose very distinct; pulse 120, active and hard; skin hot and dry; no tendency to sweating; is restless; complains a good deal of pain in the shoulder and side; has not slept much to-day. The physical signs of pneumonia in the lower lobe very marked, much more so than this morning.

Prescribed venesection to four ounces. Some degree of faintness was produced, but no very marked effect upon the pulse.

The blood had the thickest, toughest buffy coat I have ever seen upon blood taken from a child: full ¼ of an inch thick. The clot was soft, would not support its own weight on attempting to lift it out of the serum.

8, P. M. Dr. Wyckoff in consultation. After the bleeding the boy became immediately easy, and has dosed all the time since. He says his pain is all gone. He looks pale and quiet; is sweating quite freely across his chest; coughs much less than before the bleeding.

The bronchial respiration at the root of the lung is as in the afternoon; that in the lower lobe less distinct, especially at the base of the chest. Pulse 120, full; respiration 48.

To take a Dover's powder, if restless, and no antimony unless feverish.

Oct. 6th, 8, A. M. Pulse 112; respiration 40, without fever. Has had altogether the best night he has had yet. Wants to get up and have his clothes on this morning. Coughs much less. Has complained of pain in side but once since last visit, and that was on coughing.

Took one Dover's powder and one dose of antimony last night.

The bronchial respiration is still heard at the root of the lung but less clearly. The inspiratory sound is beginning to take on the character of the respiratory murmur. The crepitus redux is heard in the upper part of the lower lobe.

4, P. M. General symptoms as they were this morning, but the physical signs would appear to indicate a slight effort at exacerbation on the part of the disease; the bronchial respiration being a shade more distinct, with a disappearance of the crepitus.

Oct. 9th. The boy is fully convalescent. Pulse 92; respiration 28. He is sitting up in bed with his lap full of playthings. There has been a rapid decrease in the physical signs since yesterday; a faint bronchial respiration in the middle lobe, posteriorly, and dullness on percussion, being the only signs left of consolidation.

Oct. 12. Called to see my patient. He had slipped out of doors and was found in the street playing. A physical examination showed still a rude respiration at root of right lung, with some coarse mucous rhonchus.

Duration of the case five and a-half days. Bled on the second and fourth days. From first bleeding to confirmed convalescence, three and a-half days. From last bleeding to the same time, one and a-half days. The yielding of the disease was instantaneous on the last bleeding.

Case II. Lewis Vandalippe, aged 17 years, was taken suddenly ill Oct. 25th. I first saw him Oct. 27th. He then had fever, with a weak, almost jerking pulse of 112, and a scanty bloody viscid expectoration; respiration 24; pain in right side. He had had physic before I saw him.

I prescribed antimony in free doses.

On an evening visit I found his pulse reduced to 96, and respiration to 22. The antimony vomited him freely, and he was pale and faint; expectoration still viscid and bloody. There is no evidence, by the physical signs, of the seat of the pneumonia.

Oct. 28th. Coughed considerably during the night; was otherwise tolerably comfortable. Expectoration rusty; pulse 88. Has some febrile action.

Continue the antimony.

Oct. 29th. About dusk last night got an exacerbation of fever. He is in every respect worse this morning. Pulse 112, soft; respiration 24; expectoration viscid and more bloody; cheeks flushed. There is a distinct dullness on percussion, over the lower lobe, right lung, posteriorly, with marked feebleness of the respiratory murmur. There is neither crepitus nor bronchial respiration.

Prescribed venesection to fifteen ounces. The blood was taken in two vessels. The clot was firm in both. That in the first vessel had no buff; that in the second had a decided but thin buffy coat.

Oct. 30th. There is no improvement in any one symptom. Pulse 116, of more strength than yesterday; respiration 24; expectoration viscid and more abundant and bloody than heretofore. The physical signs largely increased in intensity, there being a clear bronchial respiration at the root of the right lung extending downward, but disappearing before reaching the base of the chest. It does not extend into the right axillæ. Percussion correspondingly flat.

I bled again to fifteen ounces. The boy looked pale, and called for water before the blood was stopped. It was taken in two vessels. The first had such a buff as yesterday's blood; that in the second vessel, all of which had trickled down the arm, had no buff. The clot in both vessels was firm and tenacious.

Oct. 31st. There has been no reäction since the bleeding yesterday; the expectoration almost entirely checked by the bleeding, and with but little blood staining it. Patient had a better night than usual. Pulse 92; respiration 22. The bronchial respiration at the root of the lung, middle lobe, is less clear than yesterday, while that in the lower lobe is louder and extends over a larger surface.

Nov. 1st. Pulse 84; respiration 20. Is very comfortable. Expectoration becoming more abundant and thinner; no blood in it. The physical signs have neither yielded nor increased, being this morning about as yesterday

Nov. 2d. Pulse 72. General symptoms all good. The physical signs have changed. There are only faint traces of the bronchial respiration; percussion still dull. Expectoration quite abundant and frothy, not bloody.

Nov. 5th. The boy is up about the house. There is rude respiration at the root of the lung, with considerable mucous rhonchus.

Duration of the disease eight days. Was bled on the fourth and fifth days. From first bleeding to full convalescence, four days; from last bleeding to same time, three days. The general symptoms in this case were at first so much those of congestive pneumonia, that it was only on the third day of my attendance that I decided to bleed him.

Doctors Lay and Wyckoff visited the patient with me at different times during his illness.

The society will please indulge me in a few remarks upon the pathology, prognosis and treatment of pneumonia in connection with the report of th chairman of the committee (Prof. Flint) upon that subject, made at its September meeting.

As the committee was appointed for the express consideration of the papers I had read before the society, I can with propriety claim their attention while I notice (if indeed it be not expected of me to do so) a few of the matters of fact and opinion which the investigations and experience of the accomplished writer of the report has led him to adopt, but to which I cannot in all points give my concurrence.

I am happy in being able to agree with him in most he says upon the pathology of pneumonia. In the classification I should go one step farther than he does. That into primary and secondary pneumonia I consider as a generic division, necessary for description. Of equal importance pathologically and therapeutically is a correct classification of the varieties. That which accords with my experience is the one into typhoid, congestive and sthenic pneumonia. The propriety and importance of this classification is made manifest by the fact that a pneumonia may be primary or secondary, and yet be either typhoid, congestive, or sthenic in its character or type.

The congestive variety I do not see described or recognized in any of the standard works on pneumonia, yet I have often heard it spoken of by practitioners of medicine.

I look upon its pathology as consisting of congestion in the lungs with the addition of an inflammatory element of a light grade. I will not attempt to give any description of the symptoms. This should only be done from recorded cases which I have not got. I will only say that its invasion is sudden, generally with a chill, followed by fever of only moderate severity. The expectoration is bloody, but not the viscid, rusty sputa of sthenic pneumonia. A marked distinction between this form and sthenic pneumonia will be noticed in the blood if any be drawn, and this distinction I look upon as pathognomonic. In congestive pneumonia the crassamentum has little or no buff, and the clot but little consistency beyond that observed in healthy blood, while in sthenic pneumonia it has a buffy coat and the clot is firm and tenacious.

In the only case of this variety I saw last summer, I took 17oz. of blood on the fifth day, which answered the above description, although the lung was sufficiently advanced in the stage of consolidation to give a well-developed bronchial respiration. I suppose it to prevail more in large cities, in hospitals, and in malarial districts, than in healthy, hilly, country villages and towns; and unless made dangerous by some complication or by malaria, I look upon it as uniformly ending in recovery.

A point in my opinion of the highest importance to a correct understanding of the pathology and treatment of pneumonia, has not been referred to in the report. I refer to the fact of the excess of fibrin in the blood so long ago pointed out by Andral and Gavarret.

In every case of sthenic pneumonia I have bled this summer this element of the blood has been greatly in excess; and this fact so fully accords with all my previous experience, that I think I am warranted in asserting, not the fact of this excess of fibrin, which has long been known, but, what has not to my knowledge been heretofore asserted, that it is so uniformly so, that within certain limits it may be made the guage and measure of the severity of the disease, and a valuable guide corroborative of other indications in the use of bloodletting.

Another question of fact which has strongly impressed itself upon my mind, is, whether in typhoid pneumonia the fibrin in the blood be not really below the normal quantity. If this should be so (of which I feel a strong assurance) the proposition could be laid down that in pneumonia, as the quantity of fibrin in the blood may be in decided excess or at about the normal standard or below it, will the disease be sthenic, congestive, or typhoid in its type.

However this may be, whether true or false, the great importance of the excess of this element of the blood in pneumonia is shown by the fact that it adds to the viscidity of the blood thereby obstructing its free and easy circulation through the capillary vessels of the lungs; that it is the source from whence the fibrinous deposits in the lungs is derived, and is therefore a direct support, a "*corps-de-reserve*," to the inflammatory action going on in the lungs; and that in some instances (if not more or less so in all) it is for days largely in excess in the blood before the lungs are sufficiently implicated to yield any of the physical signs of pneumonia; giving some probability to the idea that pneumonia may be primarily a blood disease, of which the alteration in the lungs is but the physical expression. Whether these ideas of the humoral pathology of pneumonia be or be not true (I have some proofs of their correctness, and hope, one of these days, to have more) this large excess of fibrin shown by Andral to be so common in the sthenic forms of this disease, ought not, I think, to be overlooked in the consideration of its pathology.

I would here beg to be understood that when I speak of an increased quantity of fibrin in the blood, I refer to that found in the clot as well as that in the buffy coat.

Another point in the pathology of pneumonia which I wish to notice, is its limitation and diffusion. I refer in what I say entirely to sthenic pneumonia.

It is stated in the report that "the inflammation in the adult in the great majority of cases, extends over an entire lobe at least;" and again (in the fourth proposition under the head of treatment) "that there is reason to believe that where the inflammation invades other lobes than the one originally attacked, it is due to an internal tendency which, with our present knowlledge and resources, we are unable to control."

My observations would not have led me to these conclusions. In the first place, I have almost uniformly seen pneumonia show itself first in a spot on one surface, of one lobe, of one lung, and from this spot as a centre spread centrifugally, and, in bad cases only, inwardly into the substance of the lungs. Indeed, if my observations are correct, the disposition of the disease to overleap the sulci between the lobes, and to extend itself on the periphery of an adjoining lobe, is much greater than its disposition to extend inwardly. It is very rarely indeed that I see an entire lobe affected; and the fact of its occurrence always fills me with the liveliest apprehensions for my patient's safety. But one instance of the inflammation extending over an entire lobe occurred in the eight cases I have seen this summer; and in that the extension had taken place before bleeding had been practiced. If these cases be any criterion to judge of other cases (and they fully coincide with all my previous observations) then either, it is not common for the inflammation in the adult to extend over an entire lobe; or if this be the natural tendency of the disease, then, in the early and vigorous use of the lancet, we have a resource abundantly competent, in the large majority of cases, to its limitation and control.

For the same reason, the ability to prevent, in the great majority of cases of pneumonia, the extension of the inflammation over an entire lobe even, I cannot concur in the opinion that we have no control over its diffusion from the lobe originally attacked; for, of course, the power of limiting the inflammation to a part of a lobe carries with it the question of our power of preventing its diffusion to other lobes and to the second lung.

There is another statement made in the report which I think needs some qualification. It is that "double pneumonia, *i. e.* the inflammation seated in both sides of the chest, is exceedingly rare in the adult." I know of no observations upon this point but those of M. Grisolle. From an examination of 1430 cases, he says that the inflammation was double in 18 per cent. of them, *nearly one-fifth*, a proportion certainly large enough, I think, to shake one's confidence in the opinion that pneumonia tends intrinsically to recovery.

If this be the result in cases subjected to treatment, how much larger would we naturally expect the per centage to be in the same number of cases left without treatment? I leave each one to frame his own answer.

Another question suggested by the report is, the propriety of calling the consecutive inflammation of the second lung a complication. I fully understand the writer of the report to do this; and I am not clear whether he does not also consider the implication of a second lobe as a complication.

I cannot acquiesce in the propriety of so doing, for the reason that in almost every case where two lobes or both lungs are affected, the inflammation in the beginning of the attack is for a while confined to one lobe. It is then, certainly primitive, uncomplicated pneumonia. Does, then, the extension of the inflammation, in a case, uncontrolled by treatment, change the nature of the attack and become a complication? Is it not more natural to consider the varying extent of the diffusion in different cases, as due to a like variation in the degree, force, vitality of the attack, than to refer it to an unknown internal tendency, separate and distinct from the original attack in the lobe first affected? This last opinion involves the necessity of invoking, in the explanation of the pathology of pneumonia, of two laws or forces when the sliding application of one would do just as well; one law to explain the attack in cases where one lobe only is affected, and a second to account for those where an entire lung, or both lungs, become inflamed.

It also makes necessary the unscientific course of waiting until the termination of the disease before making up an opinion as to the character and pathology of the attack; for as the diseased action may or may not extend beyond one lobe, so is or is not the case one of complicated pneumonia; is it compound or simple in its nature.

If, then, my views are correct, the term uncomplicated pneumonia ought not to be thus limited in its signification, but it should include all cases of pneumonia not complicated with inflammation of other organs, or with some adventitious circumstances not directly connected with the original attack.

To the histories of cases of pneumonia I read on a former occasion, I added as an appendix, some statistics showing the high rate of mortality from the disease. These of course included deaths without reference to age, or severity. Any mortuary table which excluded them would not give a truthful idea of pneumonia as we daily see it in children and the aged, the feeble, the destitute, the sickly, the inebriate, as well as in the middle-aged, the healthy and the temperate.

I well knew that a large proportion of the deaths from inflammation of the lungs were among children, and there was therefore a propriety in my comparing it, as I did, with some of the most fatal of the diseases of childhood. But my object was not to inquire into the influences of age, destitution and intemperance upon its mortality, but simply to show that in its totality it was one of the most fatal of diseases. The figures I gave, so far from having been directly invalidated, have among other authorities had the carefully and accurately prepared mortuary tables of England brought to their support; pneumonia in them ranking third in degree of mortality.

My colleague also directly asserts that in these statistics "the ratio of mortality ascribed to the disease is uniformly large."

But leaving the question of the general mortality of pneumonia, he states that his "present object is to endeavor to determine the intrinsic tendency of pneumonia to a fatal result disconnected from complications, associated morbid conditions and incidental circumstances," and he comes to the conclusion that primary idiopathic, uncomplicated pneumonia intrinsically tends to recovery.

This being a new position, it is right before entering upon the examination of the authorities cited for sustaining it, that I should state my own opinions. I believe that the statement or proposition is correct as to the congestive variety of pneumonia; but not entirely correct as to the typhoid and sthenic types of the disease. In what I have to say I shall, as usual, have reference entirely to sthenic pneumonia.

The author of the report arrives at the conclusion above referred to, by an indirect method of examination, that of citing the results in private and public practice, and under different methods of management. A short examination of these citations will show what degree of value should be given them for the two purposes to which they are applied; first, as showing the intrinsic tendency of pneumonia to recovery, and second, their bearing upon the subject of the treatment of pneumonia by bloodletting.

The first citation made is the author's own cases. They amount to thirty-eight. Six of them died. In two no autopsy was made, and it was not known whether they were idiopathic and uncomplicated; one had pericarditic, and three were cases of double pneumonia. By excluding all these the author concludes that he has not had an instance of death from primitive, uncomplicated pneumonia; and thence infers that primitive, uncomplicated pneumonia, confined to a lower lobe, as is the case in the vast majority of instances, invariably ends in recovery." This brings up the question before discussed, whether it is right to exclude from our calculation cases of the consecutive inflammation of the second lung where the inflammation was originally and for a time confined to one lobe, but from the severity of the attack, or want of treatment, extended itself beyond the lobe first attacked. I have already expressed my views upon this question and there is no need here of repeating them.

But however this point is decided the fact stands that of thirty-eight cases of pneumonia, six died, a mortality of one in six nearly, and thus it must stand in the mortuary and statistical tables. Does not this statement confirm rather than throw doubt upon these tables? The mortality was 16 per cent. Are we warranted in affirming that degree of fatality as compatible with the opinion of the intrinsic tendency of the disease to recovery?

The cases of Dr. Ames, of Alabama, are next cited by my colleague, to prove the light mortality of pneumonia. They were fifty-five cases with only one death, having an average duration of nine days and treated by quinine, phosphorous and tr. of aconite, and without bloodletting. The disease, we are told, prevails "in an epidemic form, associated frequently with phenomena distinguished as miasmatic." I would respectfully suggest that the fact last stated destroys the appropriateness of their citation as illustrative of the tendencies or treatment of primitive sporadic pneumonia.

The results from the British army and navy, show remarkably well. Only 3 per cent. in the former, and 4 per cent. in the latter, died of those attacked with pneumonia in those two services.

As a citation showing the small mortality from English practice in pneumonia (as all know, that of early and free bleeding) the most inveterate stickler for bleeding could ask nothing better. But if it be intended, from the small mortality, to draw the inference of the intrinsic tendency of the disease to recovery without bloodletting, I plead the *non-sequitur*. It does not follow unless it is shown at the same time that they were not bled.

The remarks just made respecting the British army and navy statistics, may also be made of Laennac's cases which are cited, as for any inference against the utility of bloodletting. His practice was, as stated in the report, to bleed once moderately and then to give antimony after the Italian method.

A fact stated by M. Louis invalidates in part the value of any deductions made from Laennac's cases, whatever the treatment or result. He says that "Laennac made his diagnosis in a certain number of cases by auscultation alone, and that he considered crepitation independently of every other local symptom to be an infallible guide; so that he must have admitted many as cases of pneumonia in whom there existed crepitation only, without rusty, semi-transparent sputa, without a more or less alteration of the respiratory sound, and any degree of dullness on percussion at the part affected. In this case he must have confounded acute pulmonary catarrh with pneumonitis."

M. Louis thinks that other physicians, since Laennac's time, have fallen into the same error, for "in no other way can we account for the fact that double pneumonitis terminating successfully, is so frequently met with by some men, and so seldom by others." I cite this as a commentary upon the fact (unless otherwise explained) of the recovery of all but one of Dr. Ames' cases of double pneumonia.

The results obtained from the Vienna Hospital have, in my opinion, a truer value in determining the natural tendency of pneumonia to recovery than any of the citations made, for no active treatment was adopted. All were limited to gum water and *tisanes* containing opium. Their fatality was a little over 9 per cent. If the cases of secondary pneumonia had been excluded and the remainder correctly classified, we should have had a statement of great value. But lacking these, and believing as I do, that a majority of them were cases of congestive pneumonia, they have no great weight with me in deciding upon the question of bloodletting. The per centage of deaths is nearly three times as great as in the English services; a difference, I think, not accounted for by the explanations made for that purpose.

There is another thing to be considered in determining the value of these cases from hospitals upon the question of bleeding in pneumonia. It is that from the peculiar depressing influences with which those who seek general hospitals in their sicknesses have been previously surrounded, their diseases have not the high frank inflammatory character of the same diseases met with in private practice. I bleed my hospital cases very rarely in comparison with what I do my private patients. Their sicknesses do not call for it, and they would not tolerate it if practised. It is for this reason that deductions from hospital practice relative to the uses of bloodletting in acute diseases are not wholly reliable in their application to private practice.

At the commencement of the discussion of the subject of the treatment of pneumonia, in the report, the following principles are stated:

1st. That in determining the importance of active interference in the management of any disease, a point, first to be considered, is the intrinsic tendency of the disease to recovery or otherwise.

2d. That as a general rule the necessity for powerful remedies in the treatment of any disease, is inversely in proportion to its tendency to recovery.

3d. That in diseases which have an intrinsic tendency to a favorable issue, the proportion of recoveries is not a test of the success of particular modes of treatment.

I am happy in giving my fullest concurrence to the above propositions; but when they are applied to the treatment of (sthenic) pneumonia by bloodletting, to show that bleeding does not contribute to recoveries, I wish, with every respect for the opinions of those who do not agree with me, to interpose an emphatic negative; for it has not yet been shown, I think, that sthenic pneumonia has an uniform intrinsic tendency to recovery. All cases of this variety of the disease should be included, not merely those in which the inflammatory action has that degree of force just sufficient to involve one lobe before spending itself.

Grisolle has shown that one-fifth, nearly, of all cases of pneumonia go to the extent of involving both lungs; and we may rationally conclude that in a still larger proportion, inflammation must have extended over an entire lung. If these facts and suppositions are as I have stated and believe, it can be affirmed neither that primary pneumonia is in the vast majority of cases confined to one lobe (that is, would be if left without any treatment) nor that it has an intrinsic tendency to recovery.

The *coup-sur-coup* system of bloodletting of Bouillaud, as marked out by him to be practised in all cases indiscriminately, is not a fair exposition of the practice of those who employ this remedy. He has not, to my knowledge, a single supporter or imitator in his own country, or in this: a fact which demonstrates the universal opinion which prevails of the extravagance of his system. I would here express my deep repugnance to any such system of formularizing the treatment of pneumonia to be practised in all cases, whatever the type of the disease. Bouillaud's extravagance in bleeding, on the one side, is only equalled, on the other, by that of those "medical heretics," as they have been called, who wholly deny the necessity or propriety of ever practising venesection in pneumonia.

The investigations of M. Louis are quoted in the report to show the limited influence of bloodletting upon the duration of pneumonia. To prove any such point it is of the first importance that the bloodletting should be properly and judiciously practised.

M. Louis's first set consisted of 78 cases, which were "all in a state of perfect health at the time when the first symptoms were developed;" and yet such was the wonderfully injudicious use of bloodletting (for no other cause can be given to account for it) that 28 died; a mortality so fearful as greatly to weaken, I think, the value of any deductions from the cases as guides either to principles or practice.

M. Louis' second set of cases had a better result. It contained 29, of which 4 died. He is evidently at a loss how to account for this great difference in the fatality; and while drawing general conclusions against the utility of bloodletting, he, curiously enough, admits that it was in part the more copious character of the early bleedings in these cases which made such a difference in the mortality.

M. Louis is correct in one thing. He apparently does not compare cases of different types; but draws his conclusions from the comparison of cases of the same variety. He does not institute comparisons between cases not bled (cases so mild as to be thought not to require it) and cases bled. He bleeds all his cases, puts those bled, on or before the fourth day, into one class, and those bled after the fourth day of the disease, into another; and as much as the average duration of the first class is less than that of the second, so much is the course of the disease shortened by the bleeding. This difference amounted in his cases to about two and three-quarter days.

All of M. Louis' cases having been bled, the results cannot, of course, be so satisfactory as though he had compared two sets of cases of the same type; those in one set treated by judicious bleeding, and those in the other allowed to take their course without the abstraction of blood.

Dr. Ames' cases are again quoted to show that at least bleeding is not essential to a brief duration of pneumonia. I would suggest that their malarial, epidemic character should preclude their application to the therapeutics of sthenic sporadic pneumonia.

The cases mentioned in the report as occurring in the Massachusetts General Hospital, amount to thirty-four. Of these 29 were bled and 5 were not bled. The cases bled lasted 13 days, and those not bled 14½ days; but as Dr. Jackson candidly observes, "this would not be representing the subject in a light sufficiently favorable to our remedy (bloodletting), for in truth, the cases in which bloodletting was not employed were much less severe than the others, taking an average on each side;" and he concludes, "the advantage derived from bloodletting in our practice is greater than that derived from the same treatment in the hands of M. Louis."

The cases of our associate, Dr. Gay, may be advantageously referred to in this connection. These were 26 cases, 15 were bled on or before the third day; in no case more than once; average amount of blood taken nearly fifteen ounces. Four were bled on the fourth and fifth days; average amount of blood taken from each, six ounces; ages, averaging from 18 to 23 years. Eight cases were not bled.

I would respectfully suggest to Dr. Gay, and to the society, that the small amount of the bleeding in those bled on the fourth and fifth days could have had only a moderate effect upon the course and duration of the disease. They may, therefore, be considered almost as mild cases as those not bled. These and the cases not bled give an average duration of 13 days; while of those bled the average duration was 8 days, or nearly one-half better.

These figures do not, any more than Dr. Jackson's, show at first sight the full benefits of bleeding in proper cases; the cases bled being more severe than those not bled, and yet convalescing in nearly half the time, showing, as Dr. Gay very justly remarks, I think, that "so far as these cases are of any worth, that venesection abridged the duration of the disease, that convalescence, instead of being retarded, is accelerated, and the proclivity to typhoid symptoms warded off."

The undeniable conclusion from these cases of Drs. Jackson and Gay, is, as it seems to me, that severe cases of pneumonia, with prompt and sufficient bleedings, have a better chance of a speedy convalescence than mild cases which are not of a character or type to justify bloodletting.

Dr. Flint compares six cases treated by him during the summer without bleeding, with four cases I bled, and correctly shows that their average duration exceeds mine by a fraction less than a day. He asks whether the abstraction of blood was necessary in my cases; "in other words, whether the favorable progress of the disease was not due to its natural tendencies, irrespective of treatment."

Now in comparing cases for the purpose of ascertaining the influence of treatment upon the duration, it is absolutely necessary, for safe and reliable deductions, that they be of the same type.

Among the cases I reported to the society in September, were two I did not bleed; but it was the last thing that occurred to me, that as these did so well without bloodletting, I was, perhaps, bleeding the others unnecessarily. The wholly different aspect of the cases forbid any such idea.

I will examine our cases in the light thrown upon the subject by the cases of Drs. Jackson and Gay, already quoted.

| Cases Bled. | Duration. | Average No. of Bleedings. | Average amount of blood taken from each patient. |
|---|---|---|---|
| Dr. Jackson's cases, - | 13 days. | 2 | 30½ oz. |
| " Gay's " - | 8 " | 1 | 15 " |
| My " - | 8¾ " | 2 | 33 " |

As far as my practice goes, the number of bleedings and the amount of blood abstracted with benefit (having due reference to the age of the patient) are always good guides by which to judge of the severity and persistence of the disease. By this rule my cases were more severe than Dr. Jackson's, which again are evidently more severe than Dr. Gay's.

I will now compare the cases not bled of Drs. Jackson, Gay and Flint. This will be fair, for it is not probable that any controlling or disturbing treatment was employed in any of them. Dr. Jackson's cases averaged 13½ days; Dr. Gay's, 13 days; and Dr. Flint's, 9⅓ days. The inference is clear that these cases of Dr. Flint's were considerably (fully one-third) milder than those of either of the other gentlemen.

The impropriety of comparing the cases bled with those not bled, of Drs. Jackson and Gay, for the purpose of determining whether the cases bled would not have convalesced just as well and quickly without the bloodletting; or for ascertaining the effect of venesection upon the duration of the cases, will, with their statement borne in mind of the mild character of the cases not bled, be very evident, I cannot but believe, to every one who carefully examines the statements I have just made. Nor do I think that any one would be justified in concluding that as the cases Dr. Gay bled convalesced in eight days, the cases he did not bleed would have had a duration equally short had they been similarly depleted.

Equally inappropriate do I consider it to be to institute similar comparisons between my cases and the six of Dr. Flint's. His are shown to be much milder than the mild cases of Drs. Jackson and Gay, while mine were probably more severe than either of the sets of severe cases.

Would Dr. Flint's cases have borne, without injury, an equal average depletion as mine did? Would the blood have shown an equally inflammatory character? Were they in fact of the same type? a necessity I have so much insisted upon in making comparisons for pathological or therapeutical inferences or deductions. All these questions must be answered in the affirmative, I submit, before any just comparison between them can be made.

It is said that bloodletting does not possess the power of arresting the progress of pneumonia. This depends upon what is meant by an arrest. If it be demanded that on venesection all local symptoms and signs shall immediately disappear, without reference to the general symptoms, then pneumonia cannot often be arrested.

But if it be asked, only, that all the general symptoms shall immediately and permanently subside, leaving the repair of the injuries done to the lung to be afterward accomplished, then it is often possible, and as if by magic, to arrest pneumonia by bloodletting.

If in case after case of pneumonia, with severe general symptoms, it is found that all these subside after two, or three or more bleedings, within three days after the first bleeding, and within the first week of the sickness, it can justly be said that they were cases of arrest.

If my ideas are correct concerning the limitation and diffusion of the inflammation, and a case can be limited by early and sufficient venesection to a portion of a lobe, where it would otherwise have invaded the entire lobe, or two lobes, or both lungs, then it is truly a case of arrest.

I think I have, many and many a time, accomplished, by bloodletting, an arrest in this meaning of the term.

If this be granted, I must at the same time have fully succeeded in abridging the duration of the disease, in limiting its diffusion, in lessening its local morbid effects, in promoting rapid convalescence, and the complete restoration to health. All these follow as corollaries to success in arresting the disease.

But, contradictory though it may seem, all these objects just stated, are not the immediate ones sought in practising venesection. The primary object in bleeding is to lessen the power of the circulation; and in the early stages of the disease this remains a primary object until the force of the heart's action be permanently reduced.

In accomplishing this we lessen the blood within the circulating system, to the reduced capacity of the lungs; and thus give rest to the diseased organs, relieve the gorged capillaries, and remove from the system some of the surplus of fibrin I have heretofore mentioned as always being present in true sthenic pneumonia. These are the proximate objects which present themselves to the mind preliminarily to the detraction of blood; and in accomplishing them is achieved the really secondary, although very important objects I have cited from the report, and which are erroneously stated, I think, as being the primary objects sought for in bleeding.

In thus advocating bloodletting in sthenic pneumonia, I wish especially to be understood as not going one step beyond the careful directions given by Wood, Swett, Watson, Marshall Hall, and Williams, in their treatises.

I grant the great power of the remedy, its liability to be misused, and the terrible effects which sometimes follow its use in cases not adapted to it. I know very well that a single injudicious bleeding may, during the few moments required for its abstraction, set a man ten years ahead in the journey of life; and that life itself may be sacrificed by copious, ill-judged bloodletting. But a physician has no right to make any such improper venesections; and a cautious, prudent one very seldom does. There are sufficient guides to its use, if observed, to prevent its misuse. It only requires a knowledge, such as every physician ought to possess, of the general course and tendencies of the disease in which it is proposed to employ it, a knowledge of the varieties and indications afforded by the pulse, and a clear idea, not only of what is expected to be obtained by bleeding, but of the manner in which it will accomplish it; and rarely, indeed, will it then happen to one to bleed, in acute diseases, not merely uselessly but unsuccessfully.

*Dr. Hamilton* asked the indulgence of the association that he might say a few words of deserved compliment to the manner in which the Committee on Pneumonia had discharged their duties. The reports had been elaborate and learned, in every way creditable to their authors; but he referred especially to the extremely gentlemanly and courteous manner in which a discussion so interesting and exciting, and involving such wide differences of opinion, had been conducted. No personalities, no recriminations had marked its course, and he considered it as creditable to the profession in the calmness and fairness which had characterized it.

*Dr. White* concurred in the remarks of Dr. Hamilton, but moved that the report should lie on the table unpublished for the present, to be called up at a subsequent time.

The motion having been seconded, Dr. White proceeded to review the discussion, and the manner in which it began. He spoke of Dr. Flint's report as covering the whole subject, and equal to anything in the language upon it. That Dr. Burwell had also reported on the same evening with Dr. Flint. That the present paper — which he willingly admitted to be an extremely able and ingenious argument — came before the association after Dr. Flint's departure for Louisville, and when he was placed in a position where he could not be here to reply, and sustain the positions he had advanced. It was then in courtesy and justice to Dr. Flint that he wished this paper to lie on the table until Dr. F.'s return, and until the subject had been rediscussed.

*Drs. Treat, Hawley* and *Newman* spoke to the motion. The general opinion expressed by these gentlemen was that the subject should be left with the secretary, who had power to publish or not, that it could not be discussed until the paper had been read, and that by publishing we should give Dr. Flint an opportunity to read and reply, with the whole argument before him. The fact of publication did not commit the association to Dr. Burwell's views, and his paper should not be considered as ending the discussion.

Dr. White withdrew his motion by permission, and in doing so stated that his objections were not made to the character of Dr. Burwell's paper; he considered it fair and courteous, but he had offered the proposition in justice to the feelings of an absent friend.

*Dr. White* then reported a case of some obscurity in diagnosis. He was called on the 5th of November, to a case in Wyoming Co., supposing it to be one of labor. Its history was as follows:

CASE. Mrs. F., aged 34 years, had borne four children. In August, 1854, she had an abortion, after which she menstruated regularly to November 8th, from which time she presented the symptoms of pregnancy until March 1st, when the menses returned and continued regular to date. During this time there was moderate abdominal enlargement, morning nausea, etc., and labor was expected last August. Pains came on Sept. 1st, were severe for one night, and she thinks the liquor amnii escaped. After this she was comfortable for six weeks, at the close of which time she sent for Dr. Amsden, of Gainesville, who found her suffering from acute peritonitis of a grave character. This Dr. A. treated successfully, but the cause of the uterine enlargement remaining obscure, Dr. White was called. He found her with the abdomen considerably distended at the hypogastrium, and tender; the pulse 120, and feeble; and the stomach irritable. On vaginal examination he found the os uteri pressing against the posterior pubic surface, and a large tumor, hard, and which felt as if it might be a child's head within the uterus, occupying the concavity of the sacrum. One practitioner who had seen the case, had endeavored to drag it away. Dr. Amsden had introduced a catheter within the uterus and found its cavity considerably enlarged.

Dr. White, after examining, introduced a trocar into the tumor. Finding pus present, he opened with a scalpel, and a large quantity of very offensive pus escaped. The result was that the tumor was lessened and the fever abated.

Dr. W. then proceeded to remark upon the diagnosis. The existence of pregnancy was not *proved* by any of the symptoms; but the totality of the symptoms, the age and experience of the patient, the cessation of the menses, and the presence of other rational signs of pregnancy, the enlargement of the uterus for a time; after which this ceased, the occurrence of fruitless labor pains, and finally the peritonitis, created a strong impression that this was a case of extra-uterine pregnancy.

*Dr. White*, after some conversation, remarked that he had just been urging Dr. Wilcox to report a case in which he (Dr. White) had been called in consultation. Dr. Wilcox declining, he would do so himself.

Case. Mrs. P., of Franklin street, a patient of Dr. Wilcox, called him to attend her in labor, at 2, A. M., of the 28th of October. The waters had drained off thirty-six hours previously. Dr. Wilcox found a presentation of the head in the first position, with the left foot lying beside it. The uterus was very firmly contracted, and he found it impossible either to turn or return the foot. The periodic pains were frequent and forcible. Dr. Wilcox feeling unwell, he called in the assistance of Dr. White, who also found turning, or the restoration of the foot, impossible. After waiting three hours he applied the forceps and delivered very readily. The child was partially asphyxiated, but was restored, and both mother and child are now doing well. The point of interest in the case was the unusual complication of presentation.

*Dr. Burwell* reported an analogous case. He was called to a German woman who had been long in labor with a head presentation. He applied the forceps, but though the head was movable to the touch, he could not deliver. Removed them, and after a time reäpplied them with the same result. The woman bearing down vigorously at the time, the cord came down. Dr. B. finding it did not pulsate, he proceeded to turn, when he found, on pushing back the head, that the foot was presenting beside it, so that the presentation was one of the head, foot, and funis. The foot was not as low as in Dr. White's case. The child was a large one, presenting no deformity.

*Dr. Rochester* presented some pathological specimens, and remarked that members would recollect that at a previous meeting he had exhibited two specimens of perforation of the intestine at the appendix vermiformis, for one of which he was indebted to Dr. White. In one of these cases perforation had occurred from impacted fœcal matter, while in the second it was consecutive to peritonitis, the perforation taking place from without inward. He had now a third specimen.

Case. James Rose, a coachman, of robust aspect, was taken sick on the 19th of October, and took oil to relieve what he supposed to be an attack of gravel, taking in all ℥iv. On the 20th he was well enough to drive the carriage down town. In the afternoon he had a sharp chill, with pain and dysuria, and again took oil and went to bed, stating that he had gravel which a cathartic would relieve. On the 21st Dr. Rochester was called to see him.

Found him suffering with spasmodic pain, the urine scanty and stained with blood. On introducing catheter found the bladder empty. There was also nausea and vomiting, with great tenderness of the abdomen, and well-marked signs of peritonitis. In reviewing the symptoms the most probable diagnosis seemed to be that he had been suffering from renal calculus, that the calculus had perforated the ureter, and the peritonitis was the result of urinary infiltration. Morph. gr. j, was given, to be repeated in an hour, and fomentations ordered. In the afternoon found him easier, and tossing about the bed, or lying on his side without the usual suffering attendant on change of position in this disease. On the 22d found his pulse diminished to 100. The morphine was continued with the addition of calomel. On the afternoon of the 23d he became collapsed and pulseless, and died that night.

*Post-mortem.* Present, Drs. Rochester and Hunt. The kidneys, ureters, and bladder were healthy. The peritoneum exhibited a grade of peritonitis more severe and extensive than either of the gentlemen had ever met before. Large patches of gangrenous peritoneum were scattered over the visceral surfaces, some of them several inches square, but the center of inflammatory action was evidently about the caput coli. The smell being extremely offensive, the abdominal contents were removed to the dissecting-room of the college for examination.

The appendix vermiformis was found firmly glued down to the caput coli, so much so that it required the knife to release its distal extremity. On turning it up, the perforation was found at the inferior surface of the junction of the appendix to the colon. *Within* the appendix was found a bean, which was the evident cause of death.

His specimen having been exhibited Dr. R. presented another of hypertrophy of the heart.

Case. A man of dissipated habits, aged 70 years, was admitted to the hospital on the P. M. of Nov. 3d. Dr. Rochester did not see him till the P. M. of the 4th, when he found him apparently suffering from asthma, and moribund, cold and pulseless. On examination found the region of cardiac dullness extending far to the right of the sternum, and presenting great dullness in an increased vertical diameter. Learned that he had had repeated attacks of inflammatory rheumatism, but had never suspected himself to have heart disease — never had palpitation. He died that night.

*Post-mortem.* Found an enormously enlarged heart weighing 46 oz., being four times the usual weight. Both sides were equally enlarged. Extensive and firm adhesions of the pericardium existed, on the right side of the heart, none on the left. All the valves were diseased and studded with ossific deposit—in no case very large—but in the auriculo-ventricular valves sufficiently great to cause regurgitation. A large deposit of ossific matter was found in the texture of the pericardium.

*Dr. Baker* inquired if the Committee on Organization should move in procuring a charter at once.

*Dr. Newman* moved that they be instructed to prepare a form of incorporation according to the act of 1848, and explained the details. *Carried.*

*Dr. Newman* suggested that the property of the association should be insured.

*Dr. Hamilton* moved that the Room Committee have power to insure. *Carried.*

And the association then adjourned.

SANFORD B. HUNT, Secretary.

